# Supplementary material for: Hippocampal projections to the anterior olfactory nucleus differentially convey spatiotemporal information during episodic odour memory
Source: Nat Commun. 2018 Jul 16;9:2735. doi: 10.1038/s41467-018-05131-6 (PMC6048034; doi:10.1038/s41467-018-05131-6)
Supplement: Supplementary file 1 — Supplementary Information [file 41467_2018_5131_MOESM1_ESM.pdf]

Supplementary Information

Hippocampal projections to the anterior olfactory nucleus differentially convey spatiotemporal information during episodic odour memory

Afif J. Aqrabawi & Jun Chul Kim

Contents

1. Supplementary Figure 1-7

2. Supplementary Table 1, 2

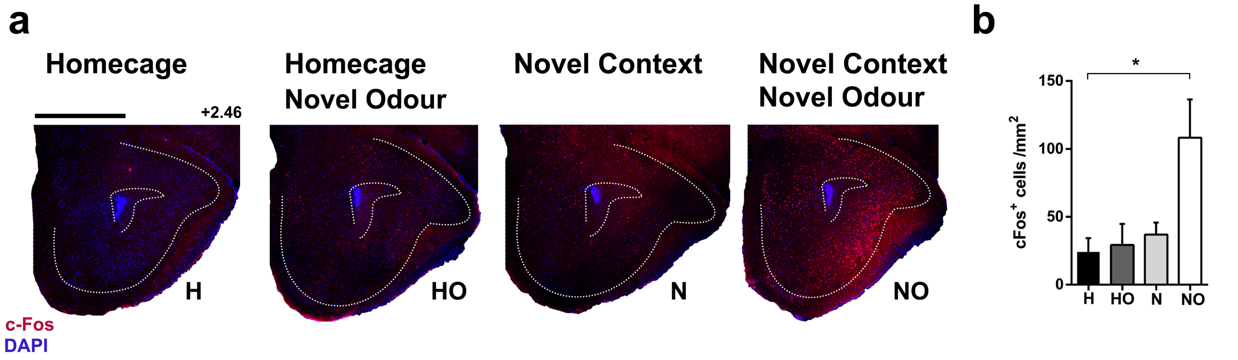

Supplementary Figure 1. Context-odour coincidence detection in the AON. (a) Using c-Fos as a proxy for neural activity, we examined the AON's response to the presence or absence of a novel context and/or a novel odour. **(b)** Animals exposed to an odour within a novel context show a significantly higher density of c-Fos positive neurons in the AON compared to homecage controls and those exposed to either stimulus separately (n=3/group; one-way ANOVA $F_{(3, 8)}$= 5.076, **P*<0.05). Data are presented as mean ± s.e.m. Scale bar represents 1 mm. Coordinate marks anteroposterior position from bregma.

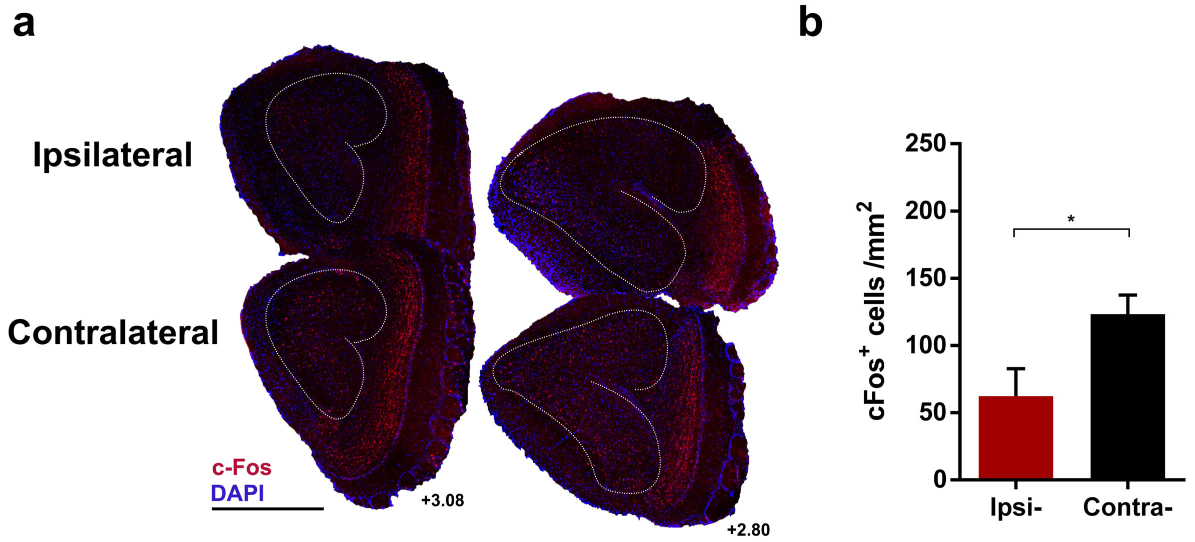

Supplementary Figure 2. Chemogenetic inhibition of AON-projecting hippocampal neurons reduces evoked activity at the AON. Animals were unilateral infused with CAV2-Cre and Cre-responsive hM4D into the AON and HPC, respectively. **(a)** Representative coronal sections demonstrating AON activity in CNO-treated mice (5 mg/kg) following exposure to a novel context-odour pairing. **(b)** A significantly lower density of c-Fos positive neurons was observed in the AON ipsilateral to the infusion site in comparison to the contralateral AON (n=4; Independent-samples t-test, $t_{(6)}$= 2.448, **P*<0.05). Data are presented as mean ± s.e.m. Scale bar represents 1 mm. Coordinate marks anteroposterior position from bregma.

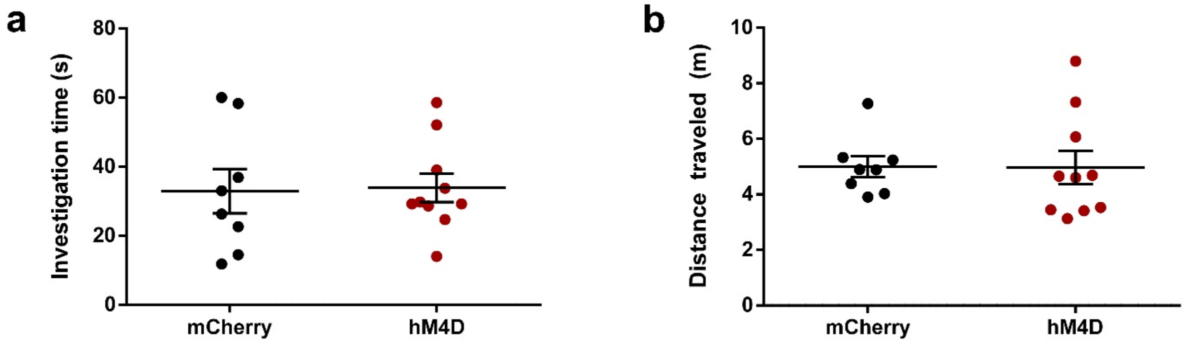

Supplementary Figure 3. Measures obtained from spatial odour memory test. (**a**) Inhibition of AON-projecting hippocampal neurons had no influence on total amount of time spent investigating odours within the context (Independent samples t-test, $t_{(16)}$=0.1322, ns, *P*=0.8964). (**b**) Total distance traveled during the retrieval phase did not differ between groups (Independent samples t-test, $t_{(16)}$=0.03624, ns, *P*=0.9715). hM4D-mCherry: n=10; mCherry control: n=8. Data are presented as mean ± s.e.m.

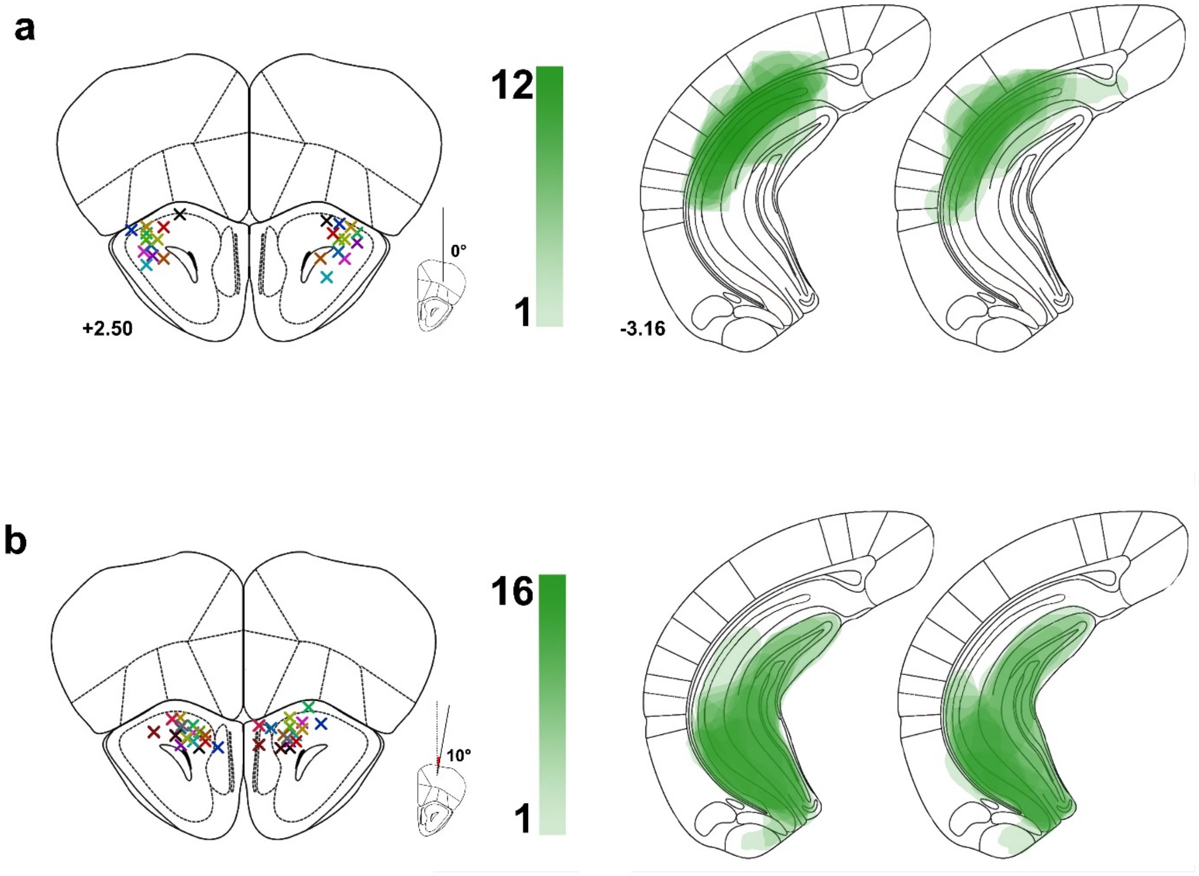

Supplementary Figure 4. Sites of optic fiber implantations in the AON and expression of ArchT in the hippocampus. (**a**) For animals in the iHPC-lAON group (n=12), optic fiber tips were predominantly located in the dorsolateral aspect of the AON (left) and AAV-mediated expression of ArchT was restricted to the intermediate HPC. (**b**) For vHPC-mAON group (n=16) optic fiber implantations were positioned above the medial aspect of the AON (left) and in most cases expression of ArchT was restricted to the CA1/Subiculum below the rhinal fissure (right). Expression was also observed in parts of the ventral CA3 and dentate gyrus, however these hippocampal subregions do not project to the AON (Aqrabawi and Kim, 2018).

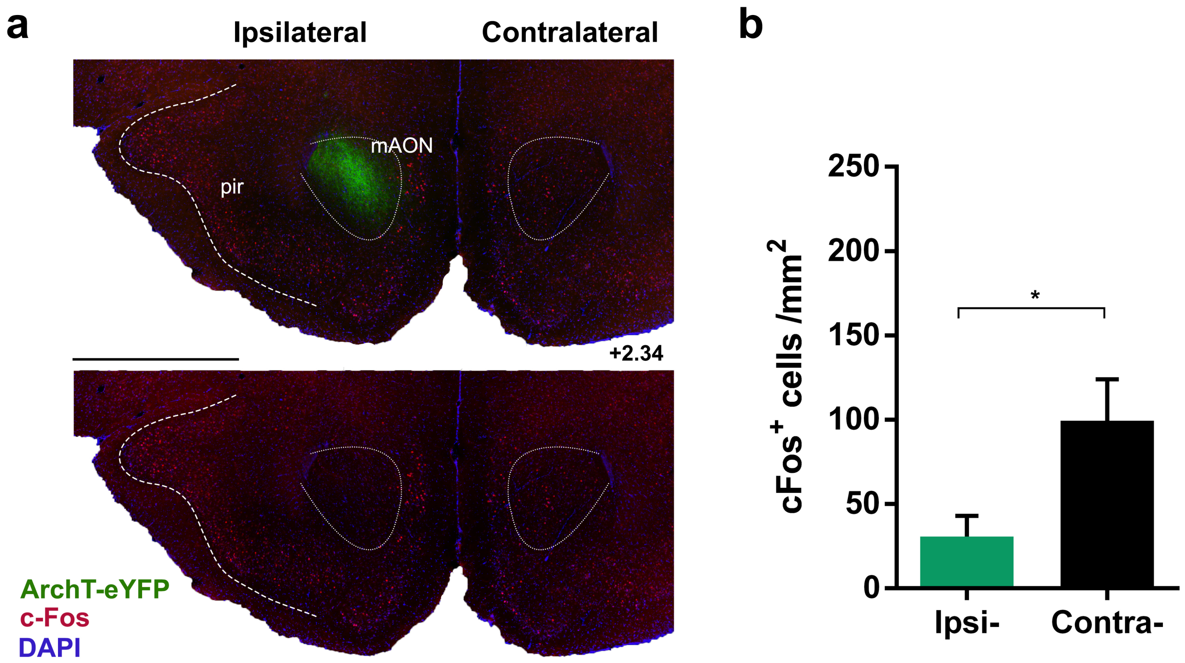

Supplementary Figure 5. Optogenetic inhibition of hippocampal terminals selectively reduces AON neural activity. Mice were unilaterally infused with AAV2/5-CaMKIIa-ArchT-eYFP into the vHPC with optic fiber implantations in the mAON and then exposed to a context-odour pairing under green light illumination (532 nm, 12 mW). **(a)** Posterior coronal section exemplifying the ipsilateral innervation of the mAON by ArchT-expressing hippocampal fibers (top) and the resulting decrease in c-Fos positive neurons in the mAON (bottom). **(b)** Cell counts of c-Fos positive neurons were conducted separately for each hemisphere, revealing a reduction in the density of active neurons within the mAON ipsilateral to the AAV-injection compared to the contralateral mAON (n=4; Independent-samples t-test, $t_{(6)}=2.505$, *$P<0.05$). Coordinate marks anteroposterior position from bregma. Scale bars represent 1 mm. mAON= medial AON; pir= piriform cortex. Data are presented as mean ± s.e.m.

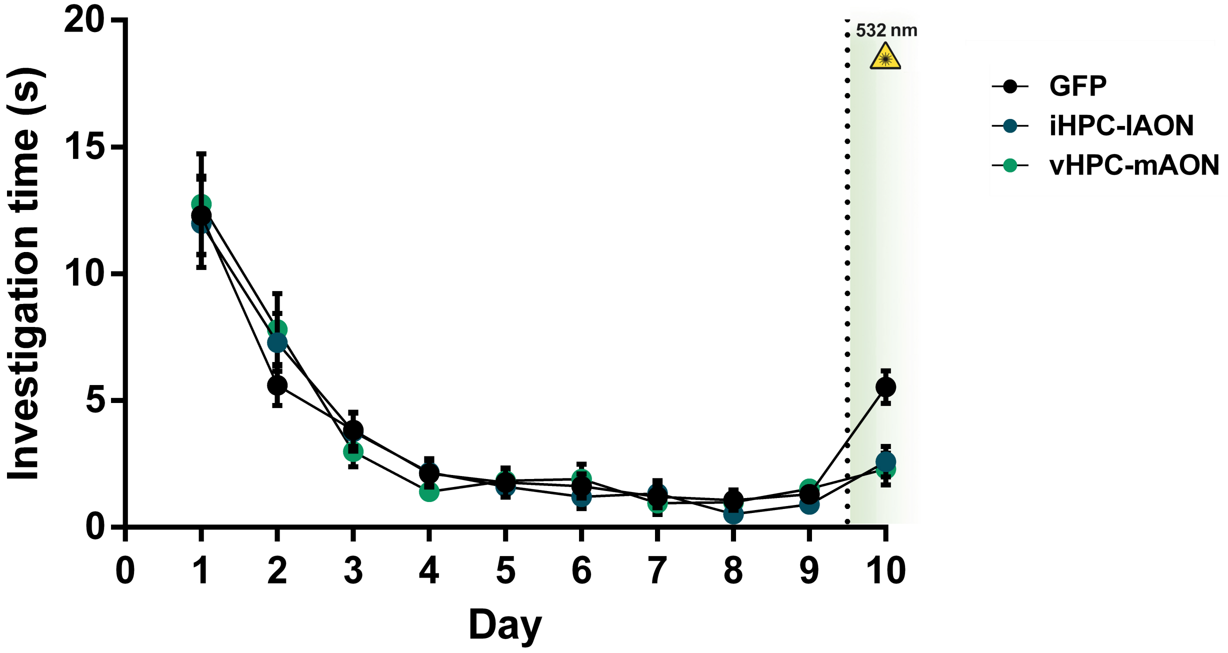

Supplementary Figure 6. Progression of odour investigation behaviour in the context-driven odour recall paradigm. Scoring the first five minutes of each exposure revealed a decrease in the time spent investigating the odour-imbued cotton swab over subsequent days of context-odour association training. When reintroduced to the context in the absence of an odour and under green light illumination (Day 10), only GFP-expressing controls displayed an increase in their investigation time compared with animals under hippocampal terminal inhibition. Data are presented as mean ± s.e.m.

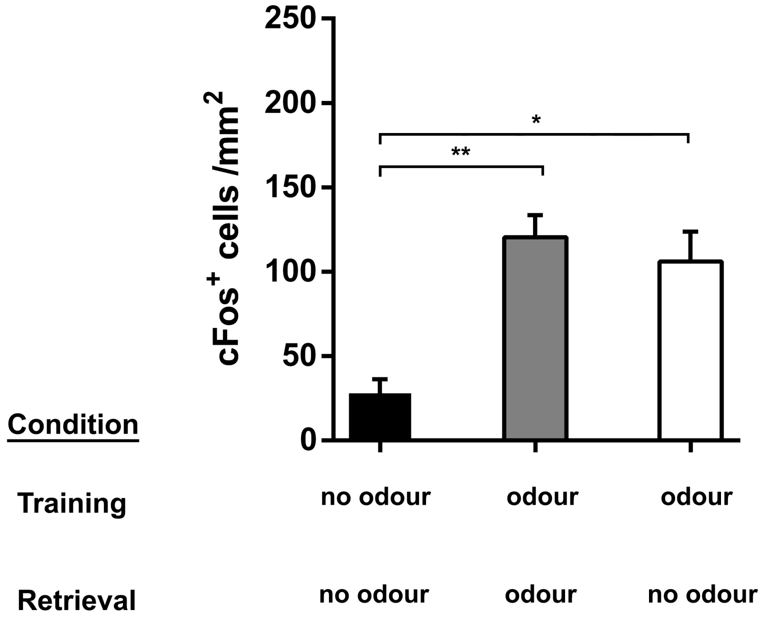

Supplementary Figure 7. Context-driven activation of AON neurons in the absence of an odour stimulus. Mice were trained on a context-odour association for 3 days and sacrificed the following day after exposure to the context-odour pair or the context alone. Both groups exhibited greater densities of c-Fos positive neurons within the AON compared to mice trained and tested with no applied odour (n=3/group; one-way ANOVA $F_{(2, 6)}$= 13.26, $**P<0.01$). Data are presented as mean ± s.e.m.

Supplementary Table 1. One-sample t-test (two-tailed) comparing discrimination ratios to zero (chance performance) for chemogenetic inhibition detailed in Fig. 1.

	Figure Number (group)					
**One sample t test**	**1c mCherry**	**1c hM4D**	**1d mCherry**	**1d hM4D**	**1e mCherry**	**1e hM4D**
Theoretical mean	0.00	0.00	0.00	0.00	0.00	0.00
Actual mean	0.39	-0.06	0.36	0.06	0.51	0.50
Std. Error of Mean	0.06	0.12	0.10	0.05	0.07	0.10
Discrepancy	-0.39	0.06	-0.36	-0.06	-0.51	-0.50
95% CI of discrepancy	0.25 o 0.53	-0.32 to 0.21	0.12 to 0.60	-0.05 to 0.18	0.35 to 0.66	0.27 to 0.73
t, df	t=6.678 df=7	t=0.4776 df=9	t=3.570 df=7	t=1.241 df=9	t=7.645 df=7	t=5.015 df=9
P value (two tailed)	0.0003	0.64	0.01	0.25	0.0001	0.0007
Significant (alpha=0.05)?	Yes	No	Yes	No	Yes	Yes

Supplementary Table 2. One-sample t-test (two-tailed) comparing discrimination ratios to zero (chance performance) for optogenetic inhibition detailed in Fig. 2.

	Figure Number (group)								
**One sample t test**	**2b GFP**	**2b iHPC-lAON**	**2b vHPC-mAON**	**2c GFP**	**2c iHPC-lAON**	**2c vHPC-mAON**	**2d GFP**	**2d iHPC-lAON**	**2d vHPC-mAON**
Theoretical mean	0.00	0.00	0.00	0.00	0.00	0.00	0.00	0.00	0.00
Actual mean	0.32	-0.17	-0.10	0.38	0.23	-0.19	0.71	0.53	0.59
Std. Error of Mean	0.04	0.09	0.08	0.12	0.09	0.07	0.04	0.08	0.07
Discrepancy	-0.32	0.17	0.10	-0.38	-0.23	0.19	-0.71	-0.53	-0.59
95% CI of discrepancy	0.22 to 0.41	-0.36 to 0.019	-0.27 to 0.073	0.11 to 0.64	0.03to 0.43	-0.34 to -0.04	0.61 to 0.81	0.35 to 0.71	0.43 to 0.75
t, df	t=7.309 df=9	t=1.978 df=11	t=1.273 df=11	t=3.075 df=1	t=2.572 df=11	t=2.638 df=15	t=16.17 df=9	t=6.586 df=9	t=8.158 df=9
P value (two tailed)	< 0.0001	0.07	0.23	0.01	0.03	0.02	< 0.0001	0.0001	< 0.0001
Significant (alpha=0.05)?	Yes	No	No	Yes	Yes	Yes	Yes	Yes	Yes
