# Supplementary material for: Hippocampal projections to the anterior olfactory nucleus differentially convey spatiotemporal information during episodic odour memory
Source: Nat Commun. 2018 Jul 16;9:2735. doi: 10.1038/s41467-018-05131-6 (PMC6048034; doi:10.1038/s41467-018-05131-6)
Supplement: Supplementary file 3 — Description of Additional Supplementary Files [file 41467_2018_5131_MOESM3_ESM.pdf]

Description of Additional Supplementary Files:

Supplementary Movie 1. Representative footage of the first minute of the context-driven odour recollection test. Left demonstrates behaviour on day 9 of the odour-context association training. Right depicts the same mouse's behaviour upon reintroduction to the context on day 10 with no odour applied to the cotton swab. A clear increase in investigation of the cotton swab is observed.
